# Trabecular Bone Deficit and Enhanced Anabolic Response to Re-Ambulation after Disuse in Perlecan-Deficient Skeleton

**DOI:** 10.3390/biom10020198

**Published:** 2020-01-29

**Authors:** Ashutosh Parajuli, Shaopeng Pei, Hongbo Zhao, Jerahme R. Martinez, X. Lucas Lu, X. Sherry Liu, Mary C. Farach-Carson, Catherine B. Kirn-Safran, Liyun Wang

**Affiliations:** 1 Center for Biomechanical Engineering Research, Department of Mechanical Engineering, University of Delaware, Newark, DE 19716, USA; parajuli@udel.edu (A.P.); shaopeng@udel.edu (S.P.); Jerahme@udel.edu (J.R.M.); xlu@udel.edu (X.L.L.); 2 McKay Orthopaedic Research Laboratory, Department of Orthopaedic Surgery, Perelman School of Medicine, University of Pennsylvania, Philadelphia, PA 19104, USA; zhhongbo@pennmedicine.upenn.edu (H.Z.);; 3 Department of Diagnostic and Biomedical Sciences, School of Dentistry, University of Texas Health Science Center, Houston, TX 77054, USA; Mary.C.FarachCarson@uth.tmc.edu; 4 Department of Biology, Widener University, Chester, PA 19013, USA; cbsafran@widener.edu; 5 Department of Biological Sciences, University of Delaware, Newark, DE 19716, USA

**Keywords:** perlecan, Schwartz-Jampel syndrome, trabecular bone, osteoporosis, osteoclastogenesis, hindlimb suspension, re-ambulation, micro-computed tomography

## Abstract

Perlecan/Hspg2, a large monomeric heparan sulfate proteoglycan, is found in the basement membrane and extracellular matrix, where it acts as a matrix scaffold, growth factor depot, and tissue barrier. Perlecan deficiency leads to skeletal dysplasia in Schwartz-Jampel Syndrome (SJS) and is a risk factor for osteoporosis. In the SJS-mimicking murine model (Hypo), inferior cortical bone quality and impaired mechanotransduction in osteocytes were reported. This study focused on trabecular bone, where perlecan deficiency was hypothesized to result in structural deficit and altered response to disuse and re-loading. We compared the Hypo versus WT trabecular bone in both axial and appendicular skeletons of 8-38-week-old male mice, and observed severe trabecular deficit in Hypo mice, approximately 50% reduction of Tb.BV/TV regardless of skeletal site and animal age. Defects in endochondral ossification (e.g., accelerated mineralization), increases in osteoclast activity, and altered differentiation of bone progenitor cells in marrow contributed to the Hypo phenotype. The Hypo trabecular bone deteriorated further under three-week hindlimb suspension as did the WT. Re-ambulation partially recovered the lost trabecular bone in Hypo, but not in WT mice. The novel finding that low-impact loading could counter detrimental disuse effects in the perlecan-deficient skeleton suggests a strategy to maintain skeletal health in SJS patients.

## 1. Introduction

Perlecan, a large monomeric heparan sulfate proteoglycan (470 kDa, ~200 nm long), is found in the basement membrane and the extracellular matrix of connective tissues [1]. Perlecan can scaffold the extracellular matrix, modulate bioavailability of heparin-binding growth factors, and separate tissue compartments [2]. Perlecan consists of five globular core-protein domains with 3–4 negatively charged glycosaminoglycan (GAG) side chains (each ~70–100 kDa) [1,2]. Global knockout of perlecan is lethal in mice; pups display severe heart, brain, and skeletal dysplasias (reviewed in [2]). In humans, complete loss of perlecan/*HSPG2* leads to Dyssegmental Dysplasias Silverman-Handmaker (DDSH) syndrome (OMIM#224410), a lethal form of neonatal short-limbed dwarfism. In contrast, Schwartz-Jampel Syndrome (SJS, OMIM#255800) patients with mutations in the *HSPG2* gene that reduce perlecan levels display a less severe phenotype with short stature, kyphosis, skeletal dysplasia, and myotonia [3,4]. SJS patients are prone to bone and cartilage loss, as perlecan deficiency is a risk factor for osteoporosis [4]. Detailed analysis of murine models resembling SJS showed irregular column organization of the growth plate, disrupted osteo-chondral junction in embryonic and postnatal bones, and accelerated mineralization, which led to shorter and wider long bones and increased bone brittleness [5].

Although the cortical bone phenotypes associated with SJS are better characterized, the impact of perlecan loss on the trabecular bone compartment is not well understood. An examination of embryonic bone development in *Hspg2* knockout mice showed a lack of tartrate-resistant acid phosphatase (TRAP)-positive cells and delayed removal of hypertrophic growth plate [6]. In contrast, 20% more osteoclasts were observed at the tibial trabecular bone in newborn perlecan-deficient mice than wild type (WT) controls [7]. The effects of perlecan deficiency on the formation and function of osteoclasts and trabecular bone remodeling appear to be age- and context-dependent. The trabecular bone phenotypes at the axial and appendicular skeletal sites are first characterized as the function of age in this study.

Emerging evidence supports the involvement of perlecan in bone adaptation to its mechanical environment. As a major component of the pericellular matrix of bone cells, perlecan acts as a mechanical sensor, allowing osteocytes to detect mechanical signals in vivo [8]. Bone normally responds to mechanical stimulation during daily physical activities by forming new bone and maintaining homeostasis, while removal of mechanical stimulation results in bone loss and deterioration of trabecular structure [9,10]. We found that deficiency of perlecan narrows the lacunar-canalicular system (LCS) [11,12], the major transport conduit system for mature osteocytes to obtain nutrients and communicate with other cells [13,14]. Our single-molecule study showed that perlecan core protein is long and strong (~71 MPa) enough to serve as a sensing tether for osteocytes to detect fluid flow [15]. Reduction in the number of perlecan-containing sensors in the LCS diminishes bone formation in perlecan-deficient mice under two-week tibial loading [16]. How perlecan-deficient trabecular bone responds to the loss of mechanical stimulation is examined in this work. The consequence associated with disuse is relevant to SJS patients, who often have reduced mobility due to comorbidity symptoms [3].

The present study focuses on the baseline phenotype of trabecular bone and its adaptation following disuse in perlecan-deficient mice. We hypothesize that perlecan deficiency would result in structural deficits and alter bone responses to disuse and re-loading. We compared the trabecular bone phenotypes of perlecan-deficient mice and age-matched WT controls at multiple axial and appendicular skeleton sites over a large age span. We also investigated the skeletal responses to hindlimb suspension and subsequent re-ambulation in perlecan-deficient mice. The overall aim is to understand the roles of perlecan/*Hspg2* in the development, maintenance, and mechanical adaptation of trabecular bone.

## 2. Materials and Methods

### 2.1. Animals

A strain of perlecan-deficient mice backcrossed onto the C57BL/6J background at the University of Delaware (Hypo) was bred to homozygosity and used as described [5,11,12]. Hypo mice were raised with standard protocols and genotyped by tail biopsy. Age-matched WT mice were either raised in our facility or purchased from the Jackson Laboratories (Maine, US). All animal protocols were approved by the Institutional Animal Care and Use Committee (IACUC) of the University of Delaware (AUP1233) and/or University of Pennsylvania (805278).

### 2.2. Axial and Appendicular Bone Phenotypes Associated with Perlecan Deficiency

#### 2.2.1. Ages and Bone Sites

Male mice with 8 to 38 weeks of age were used in this study as our previous studies identified sex-specific differences in the cortical bone phenotype [5]. The body weight, the length of femur (an appendicular skeletal site), and the curvature of the lumbar spine (an axial skeletal site) were measured in mice of various ages. The trabecular compartments in Hypo and WT mice were examined using ex vivo and in vivo μCT scanning, dynamic histomorphometry, and histology analysis. The proliferation and differentiation potentials of mesenchymal stromal cells (MSCs) and the osteoclastogenic capacity of progenitor cells in bone marrow were investigated in vitro.

#### 2.2.2. Bone Phenotyping

##### Ex Vivo µCT Imaging

Male mice at ages of 8, 15, 18, 28, and 38 weeks (WT: *n* = 6, 6, 6, 6, 3 mice; Hypo: *n* = 4, 6, 6, 4, 4 mice) were used. The right femora and spinal columns were harvested and stored frozen until they were examined. Femur length was measured using the scout view by the Scanco μCT35 scanner (Scanco Medical AG, Bruttisellen, Switzerland). Regions of interest (ROIs) were placed at: i) Distal femoral metaphysis (1.2 mm thickness, 0.6 mm proximally from the growth plate), ii) femoral mid-shaft (0.3 mm thickness), and iii) the trabecular bone of the fourth lumbar vertebral body (L4, central 60%). The scanning parameters were 6 µm^3^ voxel size, 55 kV, 800 ms integration time, and 500 projections per scan. The images were subjected to thresholding at 350/1000 for trabecular ROIs and 380/1000 for cortical ROIs, from which standard cortical and trabecular bone measurements were derived using the vendor provided software as previously reported [16,17]. The measurements include polar moment of inertia (pMOI, mm^4^), cortical bone area fraction (Ct.BA/TA = 1 − cortical porosity, %), cortical thickness (Ct.Th, mm), bone mineral density (Ct.BMD, mgHA/cm^3^), trabecular bone volume fraction (Tb.BV/TV, %), trabecular thickness (Tb.Th, mm), trabecular number (Tb.N, 1/mm), and trabecular separation (Tb.Sp, mm).

##### Spinal Kyphotic Index

A subset of animals at the ages of 8, 18, and 28 weeks (WT: *n* = 4, 6, 4 mice; Hypo: *n* = 6, 6, 5 mice) were subjected to full body x-ray imaging using the Scout view of the Scanco μCT35 scanner. A sagittal 2D image of the entire spinal column was captured, from which the kyphotic index indicating the curvature of the spine was calculated [18].

##### In Vivo µCT Imaging

A separate set of animals (4 WT and 5 Hypo male mice) were monitored longitudinally at the ages of 12, 20, 28, and 36 weeks using an in vivo scanner (vivaCT40, Scanco Medical AG, Brüttisellen, Switzerland). For each scan, mice were anesthetized with 2–4% isoflurane and held in a custom-built holder. Three ROIs (a femoral distal metaphysis of 1.2 mm thickness located immediately proximally to the growth plate, tibial mid-shaft of 0.3 mm thickness, and the central 1.2 mm segment of the L4 vertebra) were acquired at the voxel size of either 10.5 μm (for the appendicular sites) or 15 μm (for the axial site), with 200 ms integration time, 109 μA current, and 55 kVp [17]. Compared to the ex vivo scanning method, the in vivo imaging protocol used a lower resolution and less integration time, resulting in ~20 min of scan time per animal and a safe radiation exposure dosage [19]. The images were subjected to thresholding at lower levels (310/1000, 320/1000, and 350/1000 for the ROIs in the distal femur, vertebral body, and tibial mid-shaft cortical bone, respectively), and analyzed as described above.

##### Histology and Histomorphometry

A separate set of age-matched WT and Hypo mice (*n* = 3, 8-weeks-old and *n* = 2, 18-weeks-old) were injected with calcium-binding calcein (30 mg/kg body weight) ten days and three days prior to sacrifice. The right femora were harvested and processed for plastic embedding in methylmethacrylate (MMA) and dynamic histomorphometry; and the left femora for paraffin embedding and histology. For dynamic histomorphometry, bones were fixed in 70% (*v*/*v*) alcohol, dehydrated in an ascending ethanol series (80–100%, *v*/*v*), infiltrated and embedded in MMA. Mid-shaft cross-sections were cut using an Isomet diamond saw (Buehler, Lake Bluff, IL, USA) and polished down to 30-micron thickness. Frontal sections were obtained (6–8 µm thickness) using a microtome (Leica Biosystems Inc. Buffalo Grove, IL, USA). Bone labels were imaged under a fluorescence microscope to measure the dynamic histomorphometric indices including mineralizing surface (MS/BS), mineral apposition rate (MAR), and bone formation rate (BFR) for the cortical periosteal surface, cortical endosteal surface, and distal femoral secondary spongiosa, using the OsteoMeasure^®®^ software (OsteoMetrics, Inc, Decatur, GA, USA). For histology, bones were fixed in 4% (*v*/*v*) paraformaldehyde (PFA) for 48 h and decalcified in 14% (*w*/*v*) ethylene diamine tetra acetic acid (EDTA) for 3–4 weeks on a shaker at 4 °C. The distal femoral ends were embedded in paraffin for frontal sections (5 µm thickness). Slides were stained for TRAP activity using the protocol provided by the supplier (Sigma-Aldrich, St. Louis, MO, USA). The TRAP activity in the secondary spongiosa was quantified as the percentage of the trabecular bone surface covered with TRAP positive staining (termed as TRAP.S/BS). Three slides/bone with >50 µm separation and 2-3 animals per age group (8, 18, and 41 weeks) were analyzed.

#### 2.2.3. In Vitro Assays and Rescue Experiments

##### Perlecan Quantification Using Dot-Blot Immunoassay

To confirm perlecan deficiency in Hypo mice, perlecan protein levels in bone marrow extracts were measured using a dot-blot immunoassay [11]. Bone marrow was harvested from tibia and femur (*n* = 3 mice/group) by centrifugation [20]. Bone marrow was homogenized, from which a soluble protein solution was obtained with Triton X-100 (1%, *v*/*v*) and SDS (0.1%, *w*/*v*). The dot-blot immunoassay was performed using an anti-perlecan A7L6 antibody (Novus Biologicals, LLC, Centennial, CO, USA) [21] and a goat-anti-rabbit HRP conjugated secondary antibody (1:100,000 dilution, Abcam, Cambridge, MA, USA). Perlecan protein levels were quantified via densitometry using ImageJ (NIH, Bethesda, MD, USA).

##### Self-Renewal Capacity and Differentiation of Primary MSCs

Further in vitro assays were performed to investigate the proliferation and differentiation potential of MSCs harvested from bone marrows of WT and Hypo mice. Colony forming unit-fibroblast (CFU-F) assays and mineralization assays were performed using MSCs freshly harvested from femora of 5–6 weeks old male mice (*n* = 3 mice/genotype). For the CFU-F assay, primary cells were cultured in α-MEM (Invitrogen, Carlsbad, CA, USA) supplemented with 10% (*v*/*v*) fetal bovine serum (FBS, Hyclone, Logan, UT, USA) and 1% (*v*/*v*) penicillin/streptomycin (Invitrogen), seeded at low density (3 million cells), and grown in tissue culture-treated 25 cm^2^ flasks for 10 days [22]. Cultures were fixed with ethanol and stained with 1% (*w*/*v*) crystal-violet solution. The number and area of stained colonies (CFU) were quantified using ImageJ (NIH). Three independent CFU assays were performed for each isolated cell population. For the differentiation assay [5], primary MSCs were expanded and 2.5 × 10^5^ s passage cells were plated into a 6-well plate in Dulbecco Modified Eagle’s Medium (DMEM, Invitrogen), supplemented with 10% (*v*/*v*) FBS, 2 mM l-glutamine (Invitrogen), 10^−4^ M β-mercaptoethanol (Sigma-Aldrich), 5 mM β-glycerophosphate, 50 mg/mL ascorbic acid, and penicillin and streptomycin (P/S,50 ng/mL each). After 19 days of culture with the medium changed every 2–3 days, cells were fixed and stained with 2% (*w*/*v*) alizarin red solution. The individual wells were imaged and analyzed using ImageJ.

##### Osteoclastogenesis Potential of Primary Hematopoietic Progenitor Cells and Resorptive Activity of Stimulated Osteoclasts

Freshly harvested marrow from long bones was cultured in α-MEM supplemented with recombinant macrophage colony stimulating factor (M-CSF) (25 ng/mL, PeproTech, Rocky Hill, NJ, USA) for two days to expand the macrophage population. The non-adherent osteoclast progenitor cells were isolated and plated into 96-well plates (50,000 cells/well) with or without bovine dentin slices placed at the bottom of individual wells. To induce osteoclast formation, progenitors were stimulated with a receptor activator of NF-κB ligand (RANKL, 20 ng/mL, PeproTech) and M-CSF (25 ng/mL) for nine more days [23]. Osteoclasts were identified as multinucleated cells with positive TRAP staining using a kit (Sigma-Aldrich). The resorptive ability of osteoclasts was assayed by quantifying resorption pits formed on dentin slices (Immunodiagnostic Systems, Gaithersburg, MD) and stained with toluidine blue (1% *w*/*v*, *n* = 2–3 bone chips, two animals/genotype).

##### Rescue Experiments with Exogenous Heparin Treatment

To test if the GAG loss associated with perlecan deficiency accounts for the functional differences in Hypo primary cells compared with WT, exogenous heparin (Sigma-Aldrich), which is similar to the heparan sulfate GAG side chains of perlecan, were added in a culture medium [24,25]. Mineralization and osteoclastogenesis assays were performed as above, except that progenitor cells were cultured with/without heparin (20 or 200 µg/mL). Multi-nucleated osteoclasts were counted in wells and bone chips. To assess the effect of heparin on mineralization potential of MSCs, mineralization was quantified by extracting the monolayer cells with 10% (*w*/*v*) cetylpyridinium chloride and reading the absorbance at 570 nm with a plate reader [26]. Three independent repeats were performed per dose per genotype.

### 2.3. Skeletal Responses of Perlecan-Deficient Mice to Disuse and Re-Ambulation

#### 2.3.1. Hindlimb Suspension

##### Animals

Nineteen-week-old males (WT/Hypo: *n* = 10/8 mice) were subjected to three weeks of hindlimb suspension (HLS), following an established protocol [27]. In brief, hindlimbs were elevated using tape attached to the animal’s tail to produce a 30 degrees head-down tilt, which resulted in a cephalad fluid shift and avoided weight bearing by the hindquarters. Animals had free access of food and water in the specially designed cages. Age-matched male mice (WT/Hypo: *n* = 7/4 mice) with free ambulation in the cage served as ground control (GND). Body weight was measured weekly to monitor stress level. Mice with 15% loss of body weight (indication of excess stress) would be removed from the study.

##### Longitudinal In Vivo μCT Scanning

Temporal changes of trabecular bone structure at the distal femoral metaphysis were monitored using in vivo μCT imaging. One day prior to HLS, mice underwent a baseline scanning (Week 0) under isoflurane inhalation (vivaCT40, Scanco) as described above. At the conclusion of the three-week HLS, a second scan (Week 3) was taken before sacrifice. The trabecular bone parameters were compared between the two time points for the same animal, followed by the comparisons of percentage changes between the two time points ((Week 3-Week 0)/Week 0, %) among the four experimental groups (WT-GND, WT-HLS, Hypo-GND, Hypo-HLS).

##### Transcript Levels and RT-qPCR

Markers indicating the phenotype of osteoblasts [*Alpl (aka Alp)*, *Spp1 (aka Opn)*], osteoclasts [*Tnfrsf11b (aka Opg)*, *Tnfsf11 (aka Rankl)*], and osteocytes (*Sost*) were assessed by RT-qPCR as reported in our previous study [28]. Three animals per group were analyzed with *Gapdh* as the housekeeping gene. Tibiae were rapidly dissected after sacrifice, flash-frozen in liquid nitrogen, and stored in −80 °C. Whole bone samples were processed to shorten the harvesting time to ensure high quality of the RNA samples. Total RNA was isolated using the TRIzol^®®^ Reagent (Thermo Fisher Scientific, MA, USA) and RNeasy^®®^ Mini kit (Qiagen, CA, USA) following the manufacturers’ instructions. Samples were examined spectrophotometrically (NanoDrop Technologies, Wilmington, DE, USA), and the 260/280 absorbance ratios (1.9–2.2) indicated high quality. Nucleic acid extracts were treated to remove any genomic DNA contamination using the TURBO DNA-Free™ kit (Ambion, Thermo Fisher Scientific), and stored in −80 °C prior to the RT-qPCR analysis. All qPCR experiments were performed using Power SYBR^®®^ Green PCR Master Mix (Applied Biosystems, Thermo Fisher Scientific) on an Applied Biosystems Quantstudio 3 machine. Primers were custom ordered as oligonucleotides (Invitrogen). Forward and reverse sequences are listed in the Supplemental Materials (Table S1). Transcript levels relative to the housekeeping gene product (*Gapdh*) were first calculated as the differential cycle threshold values (ΔCt) for each sample, and the fold-changes (FC) were calculated using the 2^−ΔΔCt^ method to separately assess the effects of hindlimb unloading (HLS vs. GND) and genotype (Hypo vs. WT).

#### 2.3.2. Re-Ambulation Following HLS

Another experiment was performed to examine the skeletal response to re-loading after HLS. WT and Hypo male mice (15-weeks old) were assigned to one of four groups: (1) Three-week free ambulation on the ground (GND, *n* = 6/genotype); (2) three-week hindlimb suspension (HLS, *n* = 6/genotype); (3) two sessions of three-week free ambulation on the ground (GND-GND, *n* = 6/genotype); (4) three-week HLS followed by three-week re-ambulation on the ground (HLS-GND, *n* = 5 WT and 6 Hypo). Animals in the first two groups (GND, HLS) were sacrificed at the age of 18 weeks while those in the last two groups (GND-GND, HLS-GND) at the age of 21 weeks. After femurs were harvested, the distal metaphysis was subjected to ex vivo μCT scanning as described above.

### 2.4. Statistical Analysis

Data are reported/represented as the mean ± standard deviation. One-way ANOVA and post-hoc Tukey tests were performed for multiple age groups within the same genotype, where different letters denoting any two groups indicate statistical significance (*p* < 0.05, uppercase for WT; lowercase for Hypo). Student’s t-tests were used to compare age-matched WT and Hypo groups and (*) indicates statistical significance (*p* < 0.05). Paired Student’s t-tests were used to compare two time points for the same animals, while unpaired tests between different animals. JMP software was used for all the statistical analysis (JMP, Buckinghamshire, UK).

## 3. Results

### 3.1. No Pronounced Cortical Bone Phenotype was Seen in Adult Male Perlecan-Deficient Mice

As expected, the Hypo male mice showed consistently lower body weight, shorter femur length, and elevated bone mineral density (Ct.BMD) than the age-matched WT controls. In addition, Hypo mice had increasingly higher Young’s modulus than WT controls with aging (* indicates *p* < 0.05, Supplemental Figure S1). Although Hypo bone showed some initial growth impairment at eight weeks, the cross-sectional parameters of the femoral mid-shafts (Ct.pMOI, Ct.Th, and Ct.BA/TA, Figure S1B) and dynamic histomorphometry of bone formation indices (Figure S1D) did not differ from age-matched WT controls from 15 weeks onward. For reference, the phenotype of the cortical compartment is included in the Supplemental Materials.

### 3.2. Severe Trabecular Bone Deficit was Found in the Appendicular and Axial Skeletons of Male Perlecan-Deficient Mice

**Figure 1:**
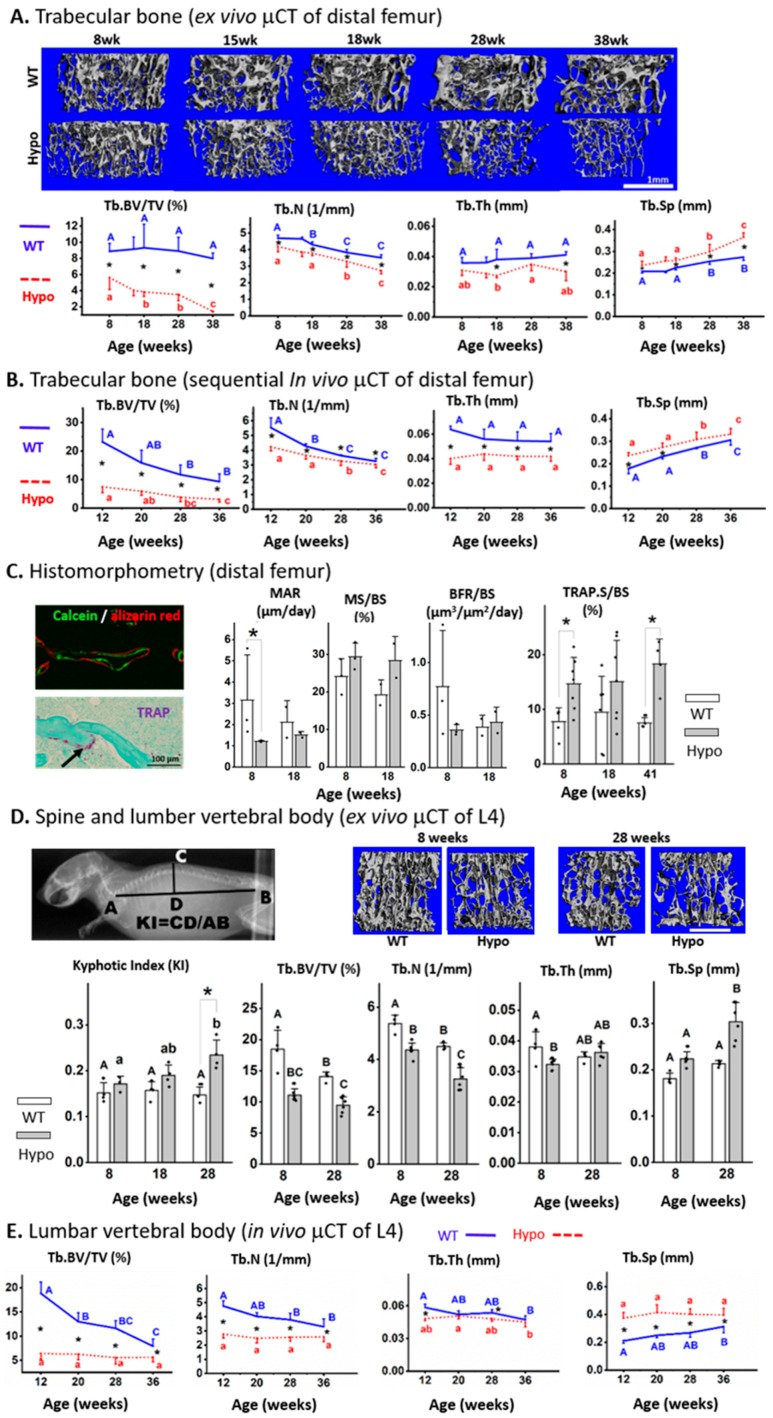
Severe trabecular osteoporosis in perlecan-deficient (Hypo) male skeletons. Distal femur (long bone) was examined (**A**) in various age groups by ex vivo μCT imaging and (**B**) in a longitudinal study using in vivo μCT imaging. (**C**) Histomorphometric and histological analysis of osteoblastic bone formation indices and osteoclastic TRAP staining. The axial skeleton (spinal column and/or the vertebral body L4) was examined (**D**) in various age groups by ex vivo μCT imaging (**E**) and in a longitudinal study by in vivo μCT imaging. Significance for within-genotype age comparisons is indicated by different letters (uppercase for WT and lowercase for Hypo) and between-genotype comparisons are indicated by * *p* < 0.05). Scale bar in Figure 1D = 1 mm.

Ex vivo µCT scans revealed a clear phenotype of trabecular bone deficit in the Hypo femurs vs. WT at all ages examined (8–38 weeks). Compared to WT, Hypo mice had reduced bone volume fraction (close or greater than 50%, Tb.BV/TV), trabecular number (~20%, Tb.N), trabecular thickness (~20%, Tb.Th), and increased trabecular spacing (>20%, Tb.Sp) (* *p* < 0.05, Figure 1A). The trabecular bone deficit was further confirmed by the in vivo µCT scans with increased statistical power due to the capability of sequentially tracking individual animals at multiple time points (Figure 1B). Temporal deterioration of the trabecular structure was detected in both genotypes as age increased from 8 to 38 weeks, which was mainly attributed to the loss of Tb.N and increased Tb.Sp (Figure 1B). Histomorphometry of the distal femur showed reduced mineral apposition rate (MAR, *p* < 0.05) in the Hypo mice at eight weeks, but no significant differences between the Hypo and WT mice in mineralizing surface (MS/BS) or the overall bone formation rate (BFR/BS) at both 8 and 18 weeks (Figure 1C). However, significantly higher TRAP-labeled surface (>100%, TRAP.S/BS) was observed in the Hypo bone at 8 and 41 weeks compared with WT (Figure 1C). As for the axial skeleton, the spinal column of the Hypo mice showed increased kyphosis with increasing age (*p* < 0.05, 8 vs. 28 weeks), and the kyphotic index of spinal curvature reached significance for Hypo mice at 28 weeks when compared with the age-matched WT (* *p* < 0.05, Figure 1D). The L4 trabecular bone in the Hypo mice showed lower Tb.BV/TV, and Tb.N (>20%) at both 8 and 28 weeks, reduced Tb.Th at 8 weeks (~15%), and increased Tb.Sp at 28 weeks (~30%, Figure 1D). The in vivo µCT scans of the same animals from 12 to 38 weeks demonstrated osteoporosis in the Hypo L4 vertebrae evidenced by their lower Tb.BV/TV, Tb.N, and Tb.Sp than was seen in the WT animals (Figure 1E). Note that the numerical values derived from the in vivo and ex vivo µCT scans differed due to different imaging/analysis settings. However, both imaging modalities consistently revealed the trabecular deficits in the Hypo skeleton.

### 3.3. Functions of Bone Progenitor Cells were Altered in Perlecan-Deficient Mice

**Figure 2:**
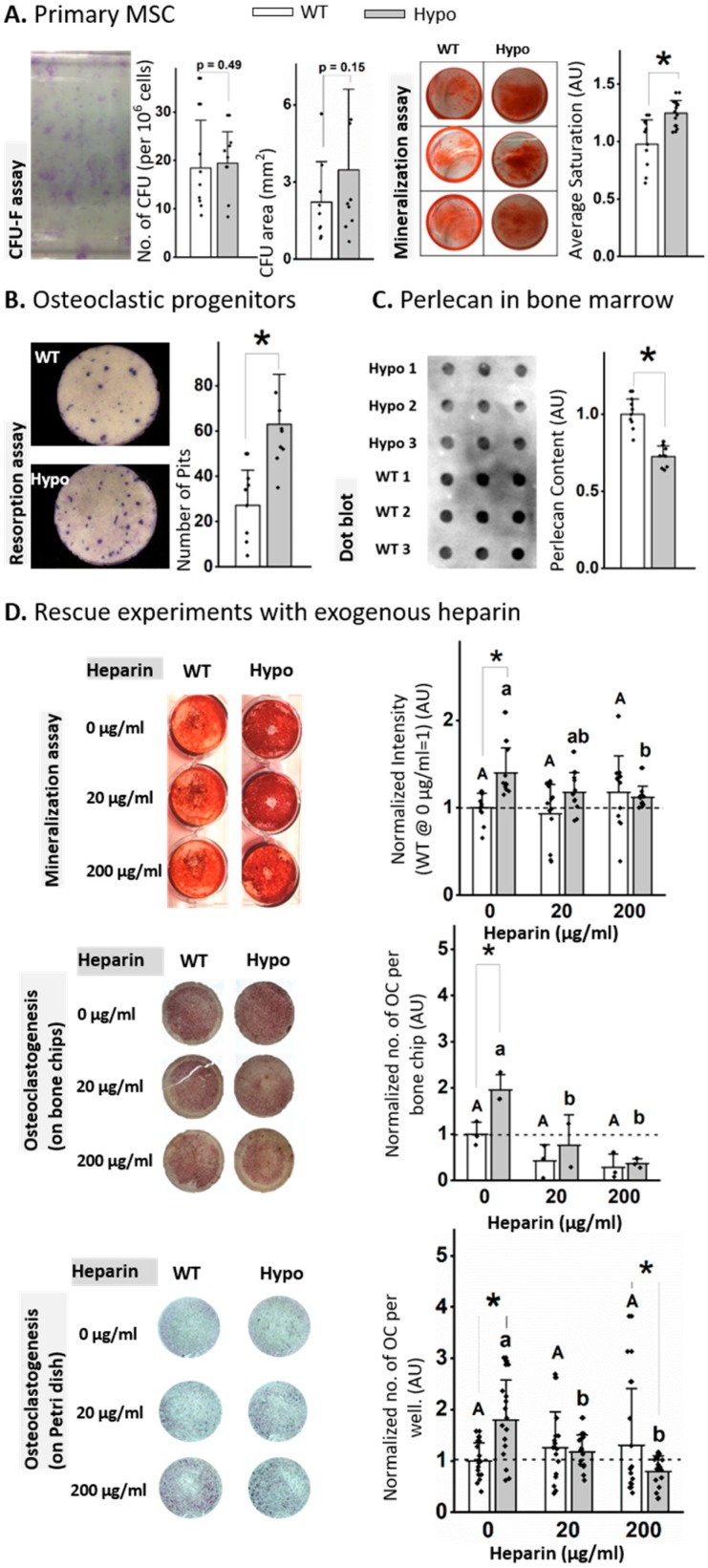
Perlecan deficiency altered MSC differentiation, but not proliferation (**A**), and increased osteoclastogenesis and resorptive activity (**B**). Perlecan content was reduced in Hypo marrow (**C**). Treating the primary MSC and osteoclast progenitor cells with exogenous heparin rescued the effects from the perlecan deficiency (**D**). Significance for within-genotype dose comparisons are indicated by different letters (uppercase for WT and lowercase for Hypo) and between-genotype comparisons indicated by * (*p* < 0.05). Open bars: WT; filled bars: Hypo.

To understand the cellular mechanisms underlying the observed trabecular deficit in the Hypo mice, we analyzed the functions of primary progenitor cells from bone marrow (Figure 2). The perlecan-deficient primary MSCs did not differ in their colony-forming capability from WT in terms of the number and size of colonies (CFU-F assays, Figure 2A). However, Hypo cells showed increased mineralization (more intensive alizarin red staining) after three-week culture in the osteogenic media (Figure 2A). As for the capacity of osteoclastogenesis, the non-adherent Hypo primary marrow cells produced more than twice resorption pits than WT counterparts when they were stimulated with two osteoclastogenic cytokines (Figure 2B). Dot blot assay of the bone marrow lysates confirmed the reduced protein level (~30%) of perlecan in the Hypo mice (Figure 2C). To discover if the GAG side chains of the perlecan regulated the progenitor cells, rescue experiments with exogenous heparin were performed on the Hypo primary cells. In the MSC mineralization assay, while the addition of heparin at the doses of 20 or 200 µg/mL did not affect the mineralization of the WT cells, the elevated mineralization in the untreated Hypo cells (*p* < 0.01) was abolished under both doses (Figure 2D). For the osteoclastogenesis assay with or without bone chips, the number of TRAP-positive cells was not affected by the addition of heparin to the WT cultures, but the increased number of TRAP-positive cells in the untreated Hypo cultures (*p* < 0.05) was abolished with the addition of either 20 or 200 µg/mL heparin (Figure 2D).

### 3.4. Trabecular Bone Responses to Three-Week Hindlimb Suspension were Similar in WT and Hypo Mice

**Figure 3:**
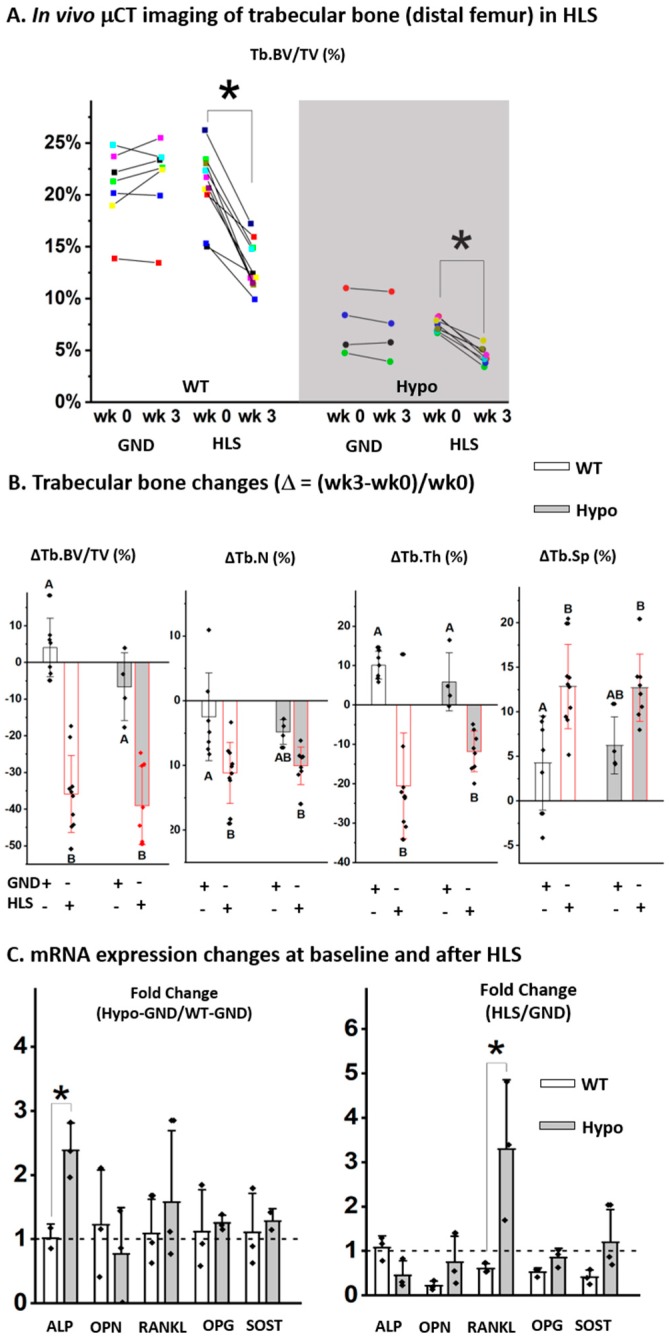
Trabecular bone responses to HLS were similar in WT and Hypo mice. (**A**) Sequential in vivo CT imaging showed bone loss after 3-weeks HLS regardless of genotype. (**B**) HLS-induced relative changes in the trabecular bone parameters were similar in Hypo and WT mice. (**C**) Fold-change of bone markers for GND control mice (Hypo-GND/WT-GND) and the fold-changes induced by HLS (HLS/GND) in both WT and Hypo mice (* *p* < 0.05, Student t test). Significance in multiple group comparisons was indicated by different letters (one-way ANOVA and Tukey post hoc tests).

In vivo sequential scanning of the distal femurs of mice showed that the trabecular bone volume fraction (Tb.BV/TV) remained largely unchanged over three weeks in the GND mice regardless of the genotype, confirming the maturity of the skeleton at the age of 19–21 weeks (Figure 3A). The results confirmed the trabecular bone deficit associated with perlecan deficiency as shown by the lower Tb.BV/TV in Hypo GND mice compared to WT controls at the start point (Week 0, Figure 3A). Three-week HLS induced a significant trabecular bone loss in both WT and Hypo mice at the end of the experiment (Week 3) compared to the baseline values (Week 0, * *p* < 0.05, Figure 3A). Percentage changes of the trabecular bone measures (∆Tb.BV/TV, ∆Tb.N, ∆Tb.Th, and ∆Tb.Sp, Figure 3B) clearly showed the deteriorated trabecular bone structure in HLS groups compared with GND controls, regardless of genotype (Figure 3B). The Hypo mice showed similar percentage changes in trabecular bone parameters to the WT mice after HLS (Figure 3B). RT-qPCR analysis of the steady-state mRNA transcript levels showed elevated *Alp* in bones from the Hypo GND mice compared to those from WT GND mice at the baseline (left panel, Figure 3C), consistent with the accelerated mineralization found for the primary MSCs (Figure 2A). Furthermore, there was a three-fold increase of *Rankl* transcript level in Hypo HLS bones relative to Hypo GND controls, which was significantly higher than that in WT bones (right panel, Figure 3C). There was no significant difference between Hypo and WT in the HLS-induced fold-changes in *ALP*, *OPN*, *OPG*, or *SOST* transcripts (Figure 3C).

### 3.5. Responses to Re-Ambulation after Hindlimb Suspension were Different in WT and Hypo Mice

**Figure 4:**
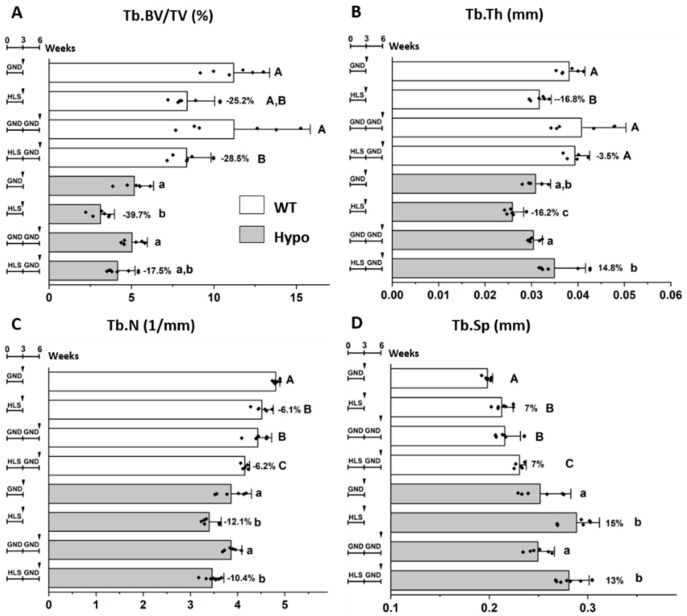
Different trabecular responses of WT and Hypo mice to re-ambulation following HLS. Tb.BV/TV was decreased by HLS and partially restored by re-ambulation in Hypo, but not WT, groups (**A**). Age and re-ambulation showed different effects on Tb.Th (**B**), Tb.N (**C**), and Tb.Sp (**D**). Significance in multiple group comparisons was indicated by different letters (uppercase for WT and lowercase for Hypo, one-way ANOVA and Tukey post hoc tests). Percentage of change is shown for the HLS group relative to its GND control ((HLS-GND)/GND, %).

Imaging of WT and Hypo mice ex vivo confirmed the comparable deterioration in major trabecular bone parameters after three weeks of HLS relative to their age-matched GND controls, including decreased Tb.BV/TV (WT/Hypo: −25.2%/−39.7%), decreased Tb.N (WT/Hypo: −6.1%/−12.1%), decreased Tb.Th (WT/Hypo: −16.8%/−16.2%), and increased Tb.Sp (WT/Hypo: 7%/15%, Figure 4). In Hypo mice, subsequent re-ambulation for three weeks partially restored Tb.BV/TV in the HLS-GND group to a level similarly found in the age-matched mice without prior suspension (GND-GND, −17.5%, *p* > 0.05), but such recovery was not seen in WT mice (HLS-GND vs. GND-GND: −28.5%, *p* < 0.05, Figure 4A). The beneficial effect of re-ambulation was evidenced by the significant increase of Tb.Th in both WT and Hypo mice (HLS-GND vs. HLS, *p* < 0.05, Figure 4B). While the Tb.Th of the re-ambulated HLS-GND group was comparable to the age-matched group (GND-GND) in WT mice (−3.5%, *p* > 0.05), Tb.Th was even higher in Hypo after re-ambulation than the age-matched group without HLS (HLS-GND vs. GND-GND, 14.8%, *p* < 0.05, Figure 4B). In contrast, during the three weeks of re-ambulation, the Tb.N and Tb.Sp continued to deteriorate in WT mice (GND-GND vs. GND and HLS-GND vs. HLS, *p* < 0.05). Such effects were not found in Hypo mice (*p* > 0.05, Figure 4C,D). Unlike its beneficial effect on Tb.Th, re-ambulation failed to improve the Tb.N, and Tb.Sp parameters for WT or Hypo mice after HLS (Figure 4C,D).

## 4. Discussion

This study revealed severe deficits in the trabecular bone compartment in the perlecan-deficient mice, which persisted in both the axial and appendicular skeletons during a life span from growth (8 weeks) to aging (38 weeks). Perlecan-deficient trabecular bone was further deteriorated under a three-week hindlimb suspension as did bone with normal perlecan expression. To our surprise, re-ambulation of the disused limbs allowed the trabecular bone to recover partially in the perlecan-deficient mice, but not in the normal mice at the age of 18 to 21 weeks. Previously, we found attenuated loading-induced cortical bone formation in perlecan-deficient mice, in contrast to the robust bone formation seen in WT mice [16]. The skeletal effects of perlecan deficiency thus are dependent on the specific (cortical vs. trabecular) bone compartment and the context of loading profiles.

The trabecular deficit shown in the perlecan-deficient skeleton is likely driven by impaired endochondral mineralization during embryonic development, altered functions of osteoblasts, osteoclasts, and their progenitor cells during postnatal bone modeling and remodeling. Perlecan core protein plays a critical role in scaffolding extracellular matrix with cell surface signaling complexes [29]. Perlecan also regulates bone growth by binding and releasing growth factors from its heparan sulfate GAG side chains. Many key controllers including fibroblast growth factors (FGFs), vascular endothelial growth factor (VEGF), RANKL, and OPG, which are important for normal functioning of cells in bone, vasculature, and muscle, are influenced directly or indirectly by the presence of heparan sulfate [2,29,30,31]. Deficiency of perlecan disorganizes the growth plate, accelerates mineralization front, and widens the bone collar during bone growth [5]. Similarly, the present study found accelerated differentiation of primary MSCs ex vivo and elevated expression of alkaline phosphatase (*Alpl*) transcripts in mature Hypo bones, which might lead to a more rapidly shrinking pool of osteoblast progenitors in bone marrow in vivo. The trabecular bone compartment, with a higher bone turnover than cortical bone compartment, is more susceptible to the shrinking pool of progenitors, resulting in impaired bone formation and a trabecular structural deficit. Elevated osteoclastic activity evidenced by TRAP staining further exacerbated the trabecular deficit phenotype in the Hypo mice. The finding that the elevated osteoclastogenesis capacity of the Hypo marrow monocytes could be normalized by the addition of heparin indicates that the increased osteoclastic activity may be partially attributed to the loss of GAG-mediated suppression of osteoclastogenesis [32]. GAGs inhibit the adherence and spreading of osteoclasts and their precursors [33], and heparan sulfate side chains of perlecan could sequester and prevent the high-mobility group box 1 protein released by bone marrow macrophages from promoting the differentiation of pre-osteoclasts [34].

The responses of perlecan-deficient skeleton to disuse and re-ambulation were somewhat surprising. Despite their baseline difference in trabecular bone volume, WT and Hypo trabecular bones showed similar degrees of bone loss (~30–40%) after three weeks of disuse, regardless of the perlecan content level. Indeed, the removal of locomotion-associated mechanical stimulation inflicted a substantial catabolic insult leading to rapid and severe bone loss. The degree of bone loss and the lack of recovery in WT skeleton during re-ambulation found in the present study were in agreement with previous reports [35]. However, partial recovery of bone loss was observed for the first time in the re-ambulated Hypo mice, and little is known about the underlying molecular mechanisms. As perlecan decorated with heparan sulfate is a potent inhibitor of mineralization [2], osteogenesis/bone formation may be enhanced in the Hypo mice where suppression of mineralization from perlecan is reduced. This partial recovery could also be due to the intrinsic coupling between bone resorption and formation processes through TGF-β [36,37]. Recently, a direct molecular coupling between osteoclasts and osteoblasts was identified through vesicular RANK released by osteoclasts [38]. Regardless of the exact molecules involved in the coupling of bone resorption and formation, elevated osteoclast activities observed in the perlecan-deficient skeleton could lead to more robust osteoblastic activities and thus enhanced recovery of lost bone after disuse in vivo. More studies are required to delineate the detailed molecular mechanisms and signaling pathways by which Hypo skeleton responds to re-loading after disuse.

The current animal model shows perlecan deficiency in the whole body, not specific to bone tissues, limiting the ability to pinpoint one tissue or cellular culprit for the observed findings. Defects in other perlecan-rich tissues/organs such as vasculature, muscle, and kidney could also contribute to the observed bone phenotype. Since the phenotype in this mouse model closely mimics human SJS, the findings presented herein could help understand the skeletal responses to mechanical environment in SJS patients. Moreover, it is noted that only males were used in the disuse and re-ambulation studies, despite sex-difference being found for the cortical bone phenotype in the mouse model [5]. Both male and female Hypo mice exhibit trabecular deficit than age-matched WT controls (Supplemental Materials). However, females in general show significantly lower baseline trabecular bone volume fraction in hindlimbs than their male counterparts, making the measurements of bone loss after disuse and bone recovery following re-ambulation technically challenging. Nonetheless, the present study revealed the severe trabecular deficits associated with perlecan deficiency, as well as the altered effects of disuse and re-loading on the perlecan-deficient skeleton. This and prior studies together demonstrate that perlecan deficiency is indeed a risk factor of osteoporosis, and that the maintenance of perlecan-deficient skeleton requires effective mechanical stimulation. Our finding that low-impact ambulation countered the detrimental disuse effects on perlecan-deficient mice suggests that physical activities could be adopted to enhance skeletal health in SJS patients.

## Supplementary Materials

The following are available online at https://www.mdpi.com/2218-273X/10/2/198/s1, Table S1: RT-qPCR primer sequences; Figure S1: No obvious cortical bone phenotype in Hypo male skeleton; Figure S2: Trabecular bone structure deficit also found in Hypo female skeleton; Figure S3: Different trabecular responses of WT and Hypo mice to re-ambulation following HLS (presented by comparing WT and Hypo for each disuse/re-loading condition).
