# Supplementary material for: Trabecular Bone Deficit and Enhanced Anabolic Response to Re-Ambulation after Disuse in Perlecan-Deficient Skeleton
Source: Biomolecules. 2020 Jan 29;10(2):198. doi: 10.3390/biom10020198 (PMC7072656; doi:10.3390/biom10020198)
Supplement: Supplementary file 1 [file biomolecules-10-00198-s001.pdf]

SUPPLEMENTAL MATERIALS

Table S1. RT-qPCR primer sequences.

Genes (Aliases)	**Forward 5'-3'**	**Reverse 5'-3'**
Alpl (Alp)	TGAATCGGAACAACCTGAC	CCACCAGCAAGAAGAAGC
Gapdh	GTGCCAGCCTCGTCCCGTAGA	TGCCGTTGAATTTGCCGTGAGT
Spp1 (Opn)	CAGCTGGATGAACCAAGTCTGGAA	ACTAGCTTGTCCTTGTGGCTGTGA
Tnfrsf11b (Opg)	GAATGCCGAGAGTGTAGAGAGGATAA	CGCTGCTTTCACAGAGGTCAAT
Tnfsf11 (Rankl)	ATCGGGAAGCGTACCTACAG	GTGCTCCCTCCTTTCATCAG
Sost	GGAATGATGCCACAGAGGTCAT	CCCGGTTCATGGTCTGGTT

1. No Obvious cortical bone phenotype in Hypo male skeleton

Both WT and Hypo mice demonstrated an overall increase in body weight and femur length with increasing age, but the Hypo mice showed consistently lower body weight and shorter femur length than the WT mice in all age groups examined (8, 15, 18, 28, and 38 weeks, * indicates $p < 0.05$, Figure S1A). While the Young's modulus derived from the three-point bending test (*Experimental set up as in [1] and data derivation as in [2]*) remained unchanged among the age groups (8–38 weeks), the Young's modulus of the Hypo femurs was significantly higher that WT at 38 weeks, despite of the lower modulus of Hypo bone at 8 weeks (Figure S1A). Ex vivo microCT scanning of the same sets of samples showed that i) the mineralization density (Ct.BMD) of the Hypo bone continued to increase with age, while that of the WT bone reached plateau at 15 weeks and older, and ii) no significant differences were found between the two genotypes in the cross-sectional structural parameters of the femoral mid-shafts (Ct.pMOI, Ct.Th, and Ct.BA/TA) at 15 weeks and

older, despite of the structural defects of Hypo bones at 8 weeks (Figure S2B). Sequential monitoring of another long bone (tibial mid-shafts) of live WT and Hypo mice showed no significant difference in the cortical structural indices (Ct.Th, Ct.BA/TA) and tissue property (Ct.BMD) between the Hypo and WT genotypes examined at most of the age points, except for the smaller Ct.pMOI at 20 and 28 weeks (Figure S1C). Similar to ex vivo imaging, a gradual increase of Ct.BMD with age was found for the Hypo tibiae as well as for the WT (Figure S1C). Dynamic histomorphometry did not find significant difference of perosteal and endosteal bone formation indices between Hypo and WT and among the age groups (15, 18, 28, and 30 weeks, Figure S1D).

Sample size: Ex vivo imaging study					
Genotype/age	8 weeks	15 weeks	18 weeks	28 weeks	38 weeks
Hypo males (# mice)	6	6	6	6	3
WT males (# mice)	4	6	6	4	4
Sample size: In vivo sequential imaging (12, 20, 28, 36 weeks of age)					
Hypo males (# mice)	5				
WT males (# mice)	4				

REFERENCES

[1] B. Wang *et al.*, “Perlecan-containing pericellular matrix regulates solute transport and mechanosensing within the osteocyte lacunar-canalicular system.,” *J. Bone Miner. Res.*, vol. 29, no. 4, pp. 878–91, Apr. 2014.

[2] J. L. Schriefer, A. G. Robling, S. J. Warden, A. J. Fournier, J. J. Mason, and C. H. Turner, “A comparison of mechanical properties derived from multiple skeletal sites in mice,” *J. Biomech.*, vol. 38, no. 3, pp. 467–475, Mar. 2005.

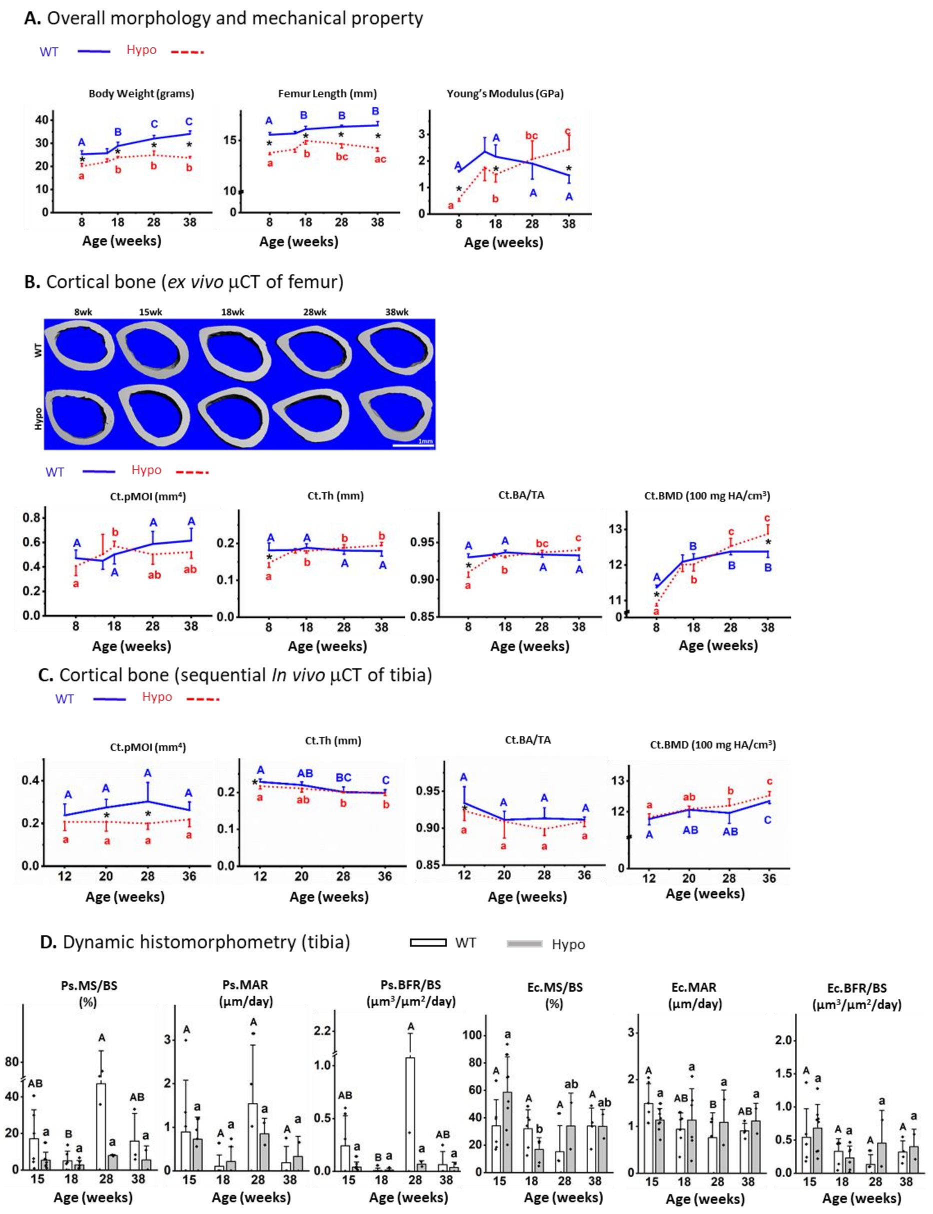

Figure S1. The effects of perlecan deficiency on cortical bone. (A) Body weight, femur length, and Young's modulus of femoral mid-shafts for WT and Hypo at different ages (8, 15, 18, 28, and 38 weeks) as in Lowe et al. (2014). (B). Cortical bone structures (Ct.pMOI, Ct.Th, Ct.BA/TA) and bone mineral density (Ct.BMD) measured at femoral mid-shafts using ex vivo microCT. (C]). Cortical bone structures sequentially measured at tibial mid-shafts using in vivo microCT. (D). Dynamic histomorphometry of bone formation indices at the periosteal and endosteal surfaces of the tibial mid-shafts. Data are presented as mean and standard deviations. One way ANOVA and post-hoc Tukey tests were performed among the different ages within the same genotype and the pairs shown with different letters (Uppercases for WT; Lowercases for Hypo) indicate statistical significance ($p < 0.05$). Student's t-tests were performed between age-matched WT and Hypo groups and (*) indicates statistical significance ($p < 0.05$).

2. Trabecular bone structure deficit also found in Hypo female skeleton

We also performed microCT scanning on female Hypo skeletons and compared them with their age-matched WT controls. Deficits of the trabecular bone parameters were noted for the Hypo females as shown in Figure S2.

Genotype/age	8 weeks	20 weeks
WT females (# mice)	4	3
Hypo females (# mice)	5	4

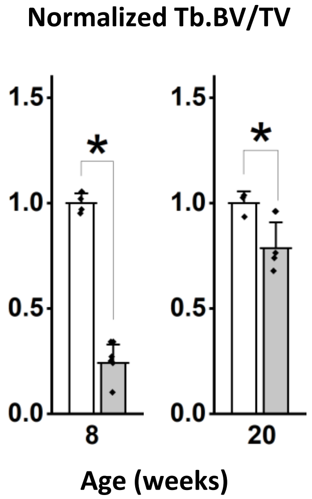

Figure S2. Similar to males, perlecan deficient females (grey bars) showed significantly lower bone volume fraction in the trabecular region of distal femur than the age-matched WT controls (white bars). Data represented as normalized Tb.BV/TV relative to WT. Statistical significance represented by * ($p < 0.05$, Student t test).

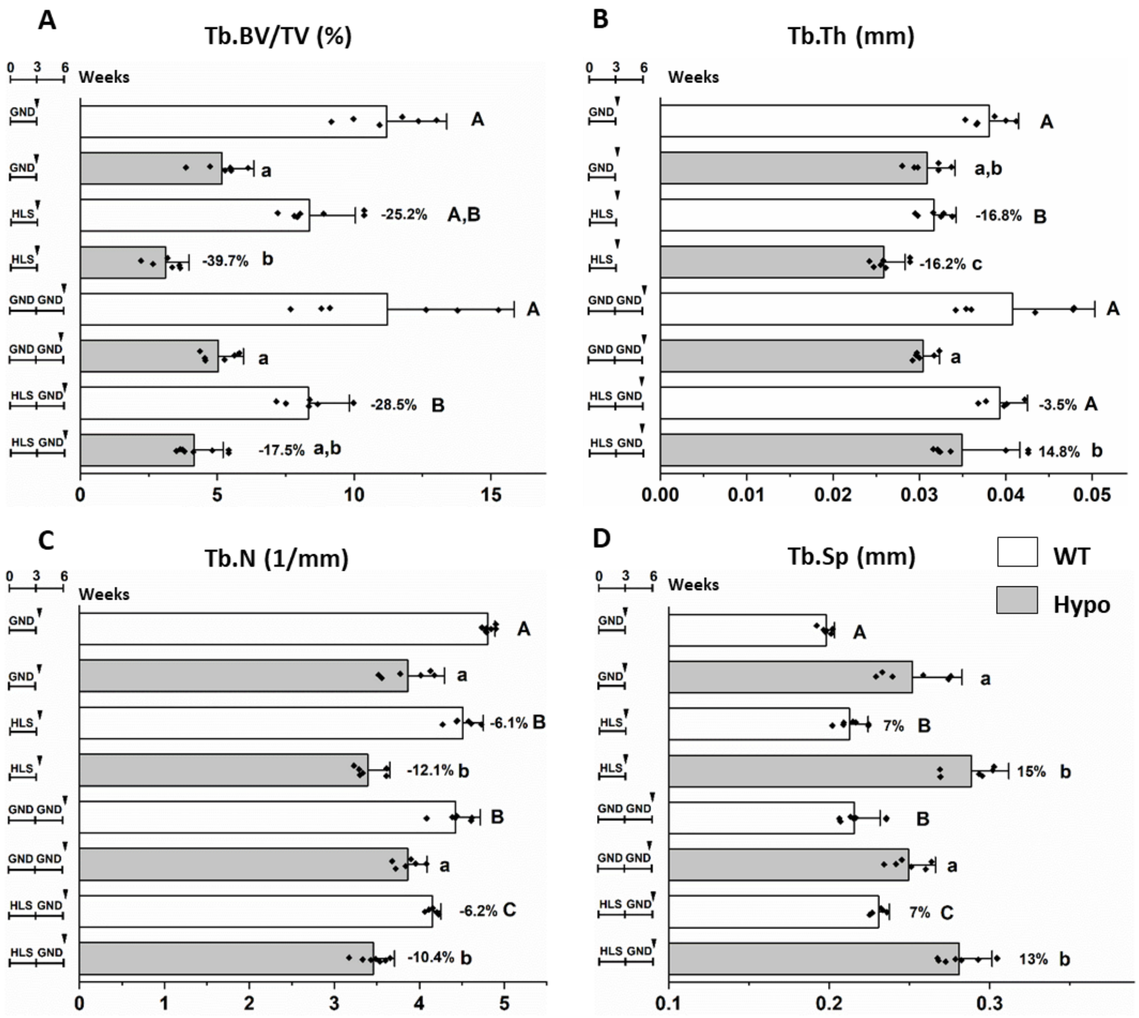

Figure S3. Different trabecular responses of WT and Hypo mice to re-ambulation following HLS. Tb.BV/TV was decreased by HLS and partially restored by re-ambulation in Hypo, but not WT, groups (A). Age and re-ambulation showed different effects on Tb.Th (B), Tb.N (C), and Tb.Sp (D). Significance in multiple group comparisons was indicated by different letters (Uppercase for WT and Lowercase for Hypo, one-way ANOVA and Tukey post hoc tests). Percentage of change is shown for the HLS group relative to its GND control ((HLS-GND)/GND, %).
